# West Nile Virus Southeast Conference^1^

**DOI:** 10.3201/eid0907.030254

**Published:** 2003-07

**Authors:** David Rimland, Jeffery Koplan, David S. Stephens

**Affiliations:** * Emory University School of Medicine, Atlanta, Georgia, USA; † Southeastern Center for Emerging Biologic Threats, Atlanta, Georgia, USA; ‡ Centers for Disease Control and Prevention, Atlanta, Georgia, USA

On January 14–15, 2003, more than 60 scientists, public health officials, and clinicians from throughout the southeastern United States gathered in Atlanta to present data from the 2001 and 2002 West Nile virus (WNV) epidemics. The aim of the daylong conference, hosted by the Southeastern Center for Emerging Biological Threats at Emory University, was to assemble a diverse perspective on WNV by sharing knowledge, identifying key questions for research about the disease and prevention, and fostering collaborations between epidemiologists, veterinarians, laboratorians, and clinicians.

In the overview presentation, the experiences of state health officials, clinical spectrum and pathogenesis, laboratory diagnostics, veterinary issues, surveillance, and vector control were discussed. The 2002 epidemic produced the largest annual number of cases to date, and four novel modes of transmission were discovered: 1) transplanted organs or tissue, including blood; 2) breast milk from an infected mother; 3) percutaneous exposure to infected tissue or serum among laboratory and hospital workers; and 4) transplacental exposure to fetuses in utero, resulting in birth defects. In addition, WNV-associated acute flaccid paralysis was discussed; the paralysis is caused by localized infection of the anterior horn cells of the spinal cord, resulting in signs and symptoms similar to poliomyelitis.

State epidemiologists or their representatives presented information about the state epidemics in the southeastern United States. The experiences in four states (Florida, Georgia, Louisiana, and Mississippi) were common in their themes of expansion of WNV epidemics, concentrated nature of outbreaks, importance of protection from mosquito bites, limitations of diagnostic methods, and dynamics of WNV spread in the United States from 2001 to 2002. Significant differences also emerged regarding the observed benefits of mosquito control activities and the value of animal surveillance as an early detection system. Also, while the states unanimously agreed that collaboration among local, state, and federal public health agencies, academic research institutions, and other nongovernmental organizations is critical to responding effectively to WNV, the degree to which such collaborations actually occurred and the existence of previously established relationships varied.

Several clinicians discussed the pathogenesis and clinical aspects of the disease. The pathogenesis of WNV was reviewed; clinicians suggested that infections of the central nervous system can demonstrate any or all of three distinct characteristics: neuroinvasiveness (the ability to enter the nervous system), neurotropism (the ability to infect neural cells), and neurovirulence (the ability to cause neurologic disease). WNV possesses all three. The virus can enter the nervous system, as shown by the encephalitis that occurs in approximately 1 in 150 infected persons; it has been shown to infect neurons; approximately 10% of patients in which the virus has invaded the nervous system eventually die. One presenter suggested that the virus had changed in the last 60 years, evolving into a more virulent strain. The neurologic manifestations of the disease were described, including weakness and flaccid paralysis (which can occur even without fever or meningoencephalitis).

WNV infection in patients with HIV was also described. Two HIV-infected patients, with CD4 cell counts below 200 cells/μL were identified with WNV by the presence of immunoglobulin M antibodies to WNV. The first was a 50-year-old homeless man co-infected with tuberculosis. Despite treatment including intravenous acyclovir, the patient continued to deteriorate, and died 18 days after admission. An autopsy revealed meningitis. The second HIV-positive patient diagnosed with WNV was a 48-year-old man who arrived at the emergency room with fever, headache, and confusion; he reported feeling “slow,” and indeed was slow to respond to questions. This patient improved rapidly and was discharged after 3 days.

The limited information about intracellular host-virus interactions was summarized. Studies in mice have identified a genetic allele that apparently confers resistance to flaviviruses. Although humans do not have a direct genetic homologue, studies to identify genetic differences to explain different clinical outcomes that should be pursued. Results of a case-control study suggested that the greatest increases in risk were related to environmental factors favoring mosquito populations. These findings in turn raised questions about factors involved in human susceptibility, risks of pesticide exposure, efficacy of mosquito control, the value of sentinel animals in surveillance, and the roles played by various species in virus transmission and amplification.

The ability to diagnose WNV in the laboratory emphasized the role of pathology, including histopathology, electron microscopy, immunohistochemistry, polymerase chain reaction, and virus isolation. Few of these tests are entirely sensitive or specific. The challenges involved in the pathologic diagnosis in animals because of the large diversity of infected species were discussed. The single most urgent concern, repeatedly emphasized by state public health officials and clinicians, is the need for a faster, simpler diagnostic test for WNV that would ease the amount of work by public health laboratories, assist physicians in correctly diagnosing infected patients, and improve surveillance by identifying subclinical cases.

The problems of vector control and the best application of these methods were also emphasized. The cost of these programs and proof of effectiveness will require careful research, including the identification of specific mosquito vectors and assessment of the long-term safety of pesticides and personal repellant applications. The most important principle in attempting emergency vector control is to consider it early, before the epidemic has evolved.

Data from in vitro studies evaluated the interaction of viral vectors and amplification hosts. These studies might elucidate the importance of birds, horses, and household pets in maintenance of epidemics. Because wild birds play a key role in the spread of WNV, the exact nature of that role must be clarified to predict the development and expansion of future epidemics.

The variation in changing epidemiology of the states’ experiences to date with WNV, even within the southeastern United States, clearly demonstrates that research needs to be replicated in numerous localities; what succeeds in one state may not prove successful in another. Whether epidemics will continue to expand in size and geographic distribution or whether a more sporadic pattern of occurrence will emerge is still unclear. Controlling WNV in the southeastern United States will take a concerted, cohesive effort. The continued collaboration of the diverse scientists in this meeting will aid in this effort. Presentations from this conference are available on the Web site for Southeastern Center for Emerging Biological Threats (available from: URL: www.secenterbiothreats.org).

